# A double blind randomized placebo controlled phase I/II study assessing the safety and efficacy of allogeneic bone marrow derived mesenchymal stem cell in critical limb ischemia

**DOI:** 10.1186/1479-5876-11-143

**Published:** 2013-06-10

**Authors:** Pawan K Gupta, Anoop Chullikana, Rajiv Parakh, Sanjay Desai, Anjan Das, Sanjay Gottipamula, Sagar Krishnamurthy, Naveen Anthony, Arun Pherwani, Anish S Majumdar

**Affiliations:** 1 Stempeutics Research Pvt Ltd, Akshay Tech Park, No. 72 & 73, 2nd Floor, EPIP Zone, Phase I-Area, Whitefield, Bangalore, 560066, India; 2 Department of Vascular Surgery, Medanta - The Medicity, Sector – 38, Gurgaon, Haryana, 122 001, India; 3 Department of Vascular and Endovascular Surgery, MS Ramaiah Memorial Hospital, MSR Nagar, MSRIT Post, Bangalore, 560054, India; 4 Consultant Vascular Surgeon, University Hospital of North Staffordshire, Newcastle-under-Lyme, Stoke-on-Trent, United Kingdom, Newcastle, ST5 0QP, UK

**Keywords:** CLI, Mesenchymal stem cells, ABPI, Bone marrow, Allogeneic

## Abstract

**Background:**

Peripheral vascular disease of the lower extremities comprises a clinical spectrum that extends from no symptoms to presentation with critical limb ischemia (CLI). Bone marrow derived Mesenchymal Stem Cells (BM- MSCs) may ameliorate the consequences of CLI due to their combinatorial potential for inducing angiogenesis and immunomodulatory environment *in situ*. The primary objective was to determine the safety of BM- MSCs in patients with CLI.

**Methods:**

Prospective, double blind randomized placebo controlled multi-center study was conducted in patients with established CLI as per Rutherford classification in category II-4, III-5, or III-6 with infra-inguinal arterial occlusive disease and were not suitable for or had failed revascularization treatment. The primary end point was incidence of treatment – related adverse events (AE). Exploratory efficacy end points were improvement in rest pain, increase in Ankle Brachial Pressure Index (ABPI), ankle pressure, healing of ulcers, and amputation rates. Twenty patients (BM-MSC: Placebo = 1:1) were administered with allogeneic BM-MSCs at a dose of 2 million cells/kg or placebo (PlasmaLyte A) at the gastrocnemius muscle of the ischemic limb.

**Results:**

Improvement was observed in the rest pain scores in both the arms. Significant increase in ABPI and ankle pressure was seen in BM-MSC arm compared to the placebo group. Incidence of AEs in the BM-MSC arm was 13 vs. 45 in the placebo arm where as serious adverse events (SAE) were similar in both the arms (5 in BM-MSC and 4 in the placebo group). SAEs resulted in death, infected gangrene, amputations in these patients. It was observed that the SAEs were related to disease progression and not related to stem cells.

**Conclusion:**

BM-MSCs are safe when injected IM at a dose of 2 million cells/kg body weight. Few efficacy parameters such as ABPI and ankle pressure showed positive trend warranting further studies.

**Trial registration:**

NIH website (http://www.clinicaltrials.gov/ct2/show/NCT00883870)

## Introduction

Peripheral vascular disease of the lower extremities comprises a clinical spectrum that extends from asymptomatic disease to presentation with critical limb ischemia (CLI). The incidence of CLI is estimated to be approximately 500 to 1000 patients per million per year [1]. Critical Limb ischemia has been defined as a condition where there is rest pain, or tissue necrosis with ulcers or gangrene in a setting of proven peripheral arterial occlusive disease [2], with an absolute ankle pressure of ≤ 70 mmHg or a toe systolic pressure of ≤ 50 mmHg [3]. About 50% patients with CLI will lose their leg within 6 – 12 months, and approximately 15% will require contralateral amputation within 2 years with one year mortality rate as high as 20% and subsequently rising to 70% and 100% at 5 and 10 years respectively [4].

Transplantation of autologous bone marrow – derived mononuclear cells (BM-MNCs) has been shown to induce neo – vascularization of ischemic tissue which introduced the concept of postnatal vasculogenesis [5,6]. Several clinical studies have shown that a variety of progenitor cell types, delivered locally by intramuscular route or systemically by intra-arterial route into the ischemic tissue exert therapeutic benefits [7-10]. More recently, using Ixmyelocel-T, an autologous expanded multicellular mixture from bone marrow, Powell et al. have demonstrated significant prolongation of time to first occurrence of treatment failure (TTF) and clinically meaningful but not significant amputation free survival time compared to the placebo treatment [11].

Bone marrow derived mesenchymal stem cells (BM-MSCs) are multipotent and differentiate easily to alternate lineages such as osteocytes, chondrocytes, adipocytes, neurons, skeletal muscle cells, endothelial cells and vascular smooth muscle cells [12-14]. BM-MSC can be expanded many folds *in vitro* and it is easy to obtain sufficient number of clinical grade BM-MSCs for cell therapy. BM-MSCS are known to secrete a number of angiogenic factors and have shown to form capillary like structures in an *in vitro* matrigel assay [15]. They also suppress lymphocyte alloreactivity in mixed lymphocyte cultures [16-18]. Because of their anti-inflammatory, immunosuppressive properties and their ability to secrete paracrine factors, BM-MSCs have been shown to be therapeutically effective in patients suffering from peripheral vascular disease.

The primary objective of this study was to determine the safety of allogeneic BM- MSCs in patients CLI by intramuscular administration. The secondary objective was to assess the efficacy of this treatment in patients with CLI.

## Methodology

### Study design

This was a phase I/II randomized, double – blind, placebo – controlled, multi-center trial. The study conformed to the Declaration of Helsinki and followed International Conference on Harmonization (ICH) – Good Clinical Practice (GCP) guidelines and was conducted with accordance to “Guidelines for Stem Cell Research and Therapy” by Department of Biotechnology and Indian Council of Medical Research (ICMR), 2007. The protocol was approved by Drug Controller General of India (Indian Food and Drug Administration) and by the Institutional Ethical Committees of the four participating hospitals in India. An Independent Data Safety Monitoring Board (DSMB) was established to assess the progress of this study and reviewed the safety data at periodic intervals. The study was registered in the NIH website (Clinical trials.gov website; http://www.clinicaltrials.gov/ct2/show/NCT00883870).

#### Criteria for enrolment

**Figure 1:**
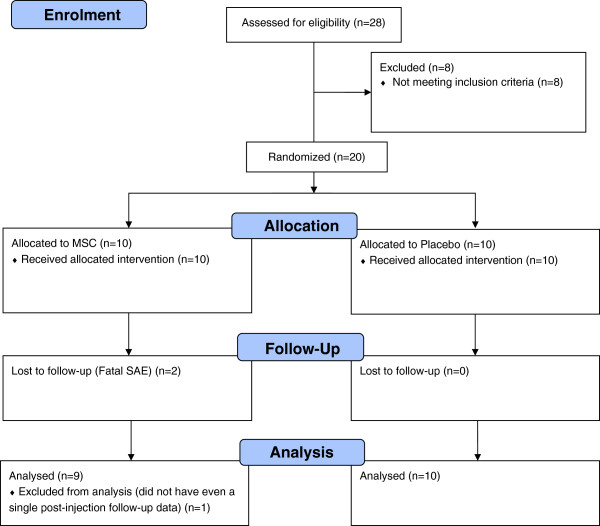
**Total patients screened, allocated in each arm, followed up and analyzed in the trial.** Diagram as per the Consolidated Standards of Reporting Trials (CONSORT) flow chart.

Patients with established CLI who had failed or were not eligible for traditional revascularization procedure were selected (Table 1). Informed consent was obtained from all patients prior to any study related activity. The detail of the total patients screened, allocated in each arm, were followed up and analyzed in the trial is given in Figure 1 (diagram as per the Consolidated Standards of Reporting Trials (CONSORT) flow chart).

**Table 1:** Eligibility criteria for patients enrolled in the study

• **Inclusion criteria**	**Exclusion criteria**
• Males or females with non-child bearing potential in the age group of 18–60 yrs. of Indian origin.	• Patients with CLI suitable for surgical or percutaneous revascularization as determined by the surgeon performing vascular procedure and patients with any acute/chronic inflammatory condition.
• Established CLI, clinically and hemodynamically confirmed as per Rutherford- II-4, III-5, or III-6; Patients having Infra-inguinal arterial occlusive disease with rest pain or ischemic ulcer/necrosis, who are not eligible for or have failed traditional revascularization treatment (No option patients).	• CLI patient requiring amputation proximal to trans-metatarsal level.
• Ankle Brachial Pressure Index (ABPI) ≤ 0.6 or ankle pressure ≤ 70 mm Hg or TcPO2 ≤ 60 mmHg in the foot.	• Patients with gait disturbance for reasons other than CLI.
• Patients if having associated Type II Diabetes should be on medication and well controlled (HbA1c ≤ 8%) without complications.	• Type I diabetes.
	• Patients having respiratory complications/left ventricular ejection fraction < 25% Stroke or myocardial infarction within last 3 months.
	• Patients who are contraindicated for MRA

### Preparation of investigational medicinal product (BM-MSC) and placebo suspension

The Investigational Medicinal Product (IMP) was BM-MSCs which obtained from bone marrow aspirates from healthy donors who were not HLA matched to the recipients. The volunteers for bone marrow donation were tested according to 21 Code of Federal Regulations (CFR) 640, FDA donor suitability & ICMR guidance for healthy bone marrow donor screening. Mesenchymal stem cells are isolated from the donor’s bone marrow mononuclear stem cells using density gradient separation method and cultured. The cells were expanded *in vitro* to manufacture the required number of cells. In the process, a donor master cell bank was maintained, which consists of cells cryopreserved in early passage which served as a source of MSCs for future manufacturing purpose. Also, a working cell bank was maintained for routine upscaling and quality control purposes. The working cell bank was upscaled further to produce the IMP at passage 4 which was used for the clinical trial. The IMP specification is given in Table 2.

**Table 2:** Investigational Medicinal Product (IMP) specification

**Description**	**Specifications**	
Morphology	Cells are fibroblastic and spindle shaped in active growing condition.	
	Cells are intact and round in shape after the trypsin action	
Cell count	180 to 220 million cells per bag	
Viability	> 85%	
Cell phenotype	CD 73 > 80%	CD 34 < 5%
	CD105 > 80%	CD 45 < 5%
	CD 90 > 80%	CD 133 < 5%
	CD 166 > 80%	CD 14 < 5%
		CD19 < 5%
		HLA-DR < 5%

Once, the desired numbers of cells have been produced, aliquots of samples were provided for quality control testing purposes. These include complete characterization by flow cytometry and differentiation capacity of these cells to osteocytes, chondrocytes and adipocytes. In addition, sterility, mycoplasma and endotoxin testing were performed at the level of MCB, WCB and IMP to confirm that the cells were devoid of any microbial contaminants and were sterile.

### Flow cytometry of BM-MSCs

For flow cytometry analysis of the surface molecule expression of BM-MSCs, the following monoclonal antibodies (Mabs) directly conjugated with fluorochromes were used: For MSC detection, CD 73-PE, CD 90-PE, CD 166-PE (Becton Dickinson, San Diego, USA) and CD 106-PE (R&D Systems, Minneapolis, USA) (positive markers {>85% positive}), while for hematopoietic cells, CD 34-PE, CD 45-FITC, CD14-FITC, CD19-FITC, HLA-DR-FITC (Becton Dickinson, San Diego, USA) and CD133-PE (Miltenyi Biotec, Gladbach, Germany) (negative markers {< 5% positive}) were used. Cells were directly coated with conjugated Mabs at room temperature for 30 mins, washed, and fixed with 1% paraformaldehyde. Next the cells were analyzed using an Easycyte Bench top flow cytometer (Guava Technologies, Millipore, MA) using Gauva Express Pro Software (Version 5.2).

### Release criteria of the BM-MSC product

Aliquots of the cells were transferred into liquid nitrogen storage vials for quality testing. Release criteria for BM - MSC administration were based on the following: (1) negative results for microbiological testing, (2) endotoxin content of < 0.06 EU/ml, (3) cell viability (trypan blue exclusion test) of > 85%, (4) pH between 7.2 to 7.4, (4) Exhibiting normal DNA ploidy (5) Normal Karyotype (6) Phenotypic marker analysis by flow cytometry as above and (7) confirmation of differentiation of cells to osteocyte, chrondocyte and adipocyte.

### Infusion parameters & treatment groups

200 million BM-MSCs in 15 ml cryopreservation medium or 15 ml PlasmaLyte A in the cryocyte bag (placebo) were thawed and re-suspended in 35 mL of PlasmaLyte A resulting in 50 mL of suspension. The IMP administration was performed under IV sedation by Midazolam and Fentanyl (12 patients) or under spinal/epidural anesthesia (8 patients) and cardio-respiratory monitoring. The constituted volume of the suspension was injected intramuscularly into the gastrocnemius muscle of ischemic lower limb (40 – 60 sites, distributed in an area of 10 cm × 6 cm, 1 – 1.5 cm in depth and 0.5 ml - 1.0 ml of BM-MSCs or placebo per site) by blinded 3 ml syringes. During the dosing, oxygen saturation was monitored 30 min prior to and 6 hrs post injection of BM-MSCs/placebo. Investigators were instructed to stop the injection if the patient showed tachypnea, cyanosis, breathlessness, or if oxygen saturation decreased to less than 85%. The patients remained in the clinical facility under supervision for at least 24 hours post IMP administration. On discharge, patients were given standard protocol of care for CLI as per investigator’s discretion. This included but not limited to analgesics, antiplatelets and anticoagulants.

A total of 28 patients were screened for enrollment in the trial, of which 20 subjects were included in the study, 10 patients each in the BM-MSC arm and placebo group respectively. All twenty patients in each of the cohorts met identical inclusion and exclusion criteria. Block randomization was conducted in a centralized manner and communicated to the Investigational Medical Product Management Team. Subjects were randomly assigned in a double-blind fashion to each group in a 1:1 ratio of BM-MSC arm to placebo. A total of five patients had presented only with rest pain (3 in BM-MSC arm and 2 in placebo arm), 10 patients presented with minor tissue loss (5 each in BM-MSC and placebo arm) and 5 patients presented with major tissue loss (3 in BM-MSC arm and 2 in placebo arm) (Table 3). An Independent Data Safety Monitoring Board (DSMB) was established to assess the progress of this study. The DSMB was comprised of Patient safety expert, Pharmacovigiliance and Pharma regulatory expert, Vascular surgeon and Statistician. They reviewed the safety data at periodic intervals, and gave their recommendations on whether to continue, modify or stop the trial. The DSMB reviewed the safety data of first five patients at the end of 1, 4 and 12 weeks after IMP administration

**Table 3:** Demographic and baseline disease condition

**Patient No.**	**Arm**	**Age (years)**	**Height (cm)**	**Weight (kg)**	**Smoking/Tobacco**	**No. of cigarettes/Chews**	**Revascularization details**	Grade of disease*
S001	Placebo	35	161	56	Past tobacco chewer	6	Right transfemoral proximal & distal thromboembolectomy with proximal tibial artery	III6
S004	Placebo	37	165	67.2	Ex-Smoker	24	No	II4
S005	BM-MSC	40	171	70.2	Ex-Smoker	10	No	III5
S007	BM-MSC	43	169	65	Ex-Smoker	48	Bypass surgery in left femoral artery	II4
S008	Placebo	32	166	42	Ex-Smoker	24	No	III5
S009	BM-MSC	46	169	60	Ex-Smoker	24	No	III6
S010	BM-MSC	57	160	42	Ex-Smoker	48	Femoropopliteal bypass above the knee	II4
S102	Placebo	45	174	72	Ex-Smoker	25	Right common femoral artery to anterior tibial artery reversed Gsv bypass	III5
S104	BM-MSC	44	159	63	Smoker	45	No	III5
S106	Placebo	32	164	71	Ex-Smoker	23	No	III5
S301	Placebo	43	167	62	Ex-Smoker	4	No	III6
S302	BM-MSC	37	165	62	Ex-Smoker	20	No	III6
S303	BM-MSC	53	172	69	Ex-Smoker	20	No	II4
S304	Placebo	59	162	60	Ex-Smoker	15	No	III6
S305	Placebo	57	167	70	Ex-Smoker	15	Right popliteal artery exploration	II4
S306	BM-MSC	46	170	46	Ex-Smoker	20	No	III5
S307	Placebo	54	165	50	Ex-Smoker	50	Femoropopliteal artery bypass above knee	III5
S308	BM-MSC	41	157	50	Ex-Smoker	20	No	III5
S402	BM-MSC	60	170	55	Ex-Smoker	23	Femoro - popliteal bypass	III5
S403	Placebo	36	167	60	Ex-Smoker	5	No	III5

*Grade of disease classified according to Rutherford criteria.

### Clinical assessments

All clinical and laboratory data, for determining both safety and efficacy parameters, were prospectively collected, and follow – up visits were performed at 1 day before and 7 days, 4 weeks, 12 weeks, 24 weeks after IMP administration. Further safety parameters were assessed after 1 year and 2 year of administration of IMP. All patients have completed two year follow-up. The study parameters were unblinded after six months follow up. Primary safety assessments included monitoring and recording of all adverse events (AEs), and assessment of electrocardiogram (ECG) parameters. Hematological and biochemical values, regular vital sign measurements, and physical examination reports were recorded in all cases. The efficacy end points included – relief of rest pain, healing of necrosis and ulceration, increase in ankle pressure, increase in ankle brachial pressure index (ABPI) and prevention of amputation in the target limb. Rest pain scores on rating scales ranged from 0 of the best (completely resolved) and 4 points for the worst condition (severe pain unresolved with paracetamol or non-steroidal anti-inflammatory drugs) [19]. Healing of all necrosis and ulcerations in the target limb was assessed by independent physician and documented by photography at each visit. Resting ABPI was measured by a Laser Doppler according to published protocol. The number of amputations was counted at the end of the study, with a focus on any potential adverse effects resulting from the BM-MSC administration during follow – up.

In addition to the routine safety laboratory parameters, levels of a few pro inflammatory cytokines were measured to evaluate the immunological response of the IMP, and they included – Interleukin – 2 (IL-2), Tumour necrosis factor (TNF) - alpha, Interferon – gamma. The estimation of these cytokines was performed by ELISA kits (abcam, San Francisco, CA, USA) using serum samples. In addition, lymphocyte profile before and after IMP injection at different time intervals was done by flow cytometry in terms of CD4, CD8 and CD25.

### Data collection

All data were recorded on manual case record forms (CRF) and verified by comparison with source documentation by third – party medical monitors. Safety assessments were performed based on the frequency of AEs and on clinically significant abnormal laboratory values. The AEs incidents are summarized as the number and percentage of subjects experiencing AE within each treatment arm.

### Statistical methods

The SAS® package (SAS® Institute Inc., USA, Version 9.2) was used for statistical evaluation. All data are presented as mean ± SD. Rest pain scale (+4 to 0) over subsequent visits were analyzed and compared between the two arms using Kruskal Wallis test. Statistical significance was defined as a two-sided p-value < 0.05. For ABPI, change from baseline to 6 months was analyzed using an analysis of variance (ANOVA) model with factors for baseline, treatment and also compared between the two arms using Kruskal Wallis test. Statistical significance was defined as a two-sided p-value < 0.05. Number of amputations, healing of ulcer and necrosis were summarized descriptively as the data was insufficient to do a statistical analysis. The primary efficacy analyses were performed in the modified intent to treat (MITT) set that included 19 patients.

## Results

### Characteristic of patients

Demographic and baseline patient data are listed in Table 3. All the patients were of Peripheral arterial disease (PAD) and the underlying etiology was either due to atherosclerosis or Thromboangitits Obliterans (TAO). Out of a total of 28 patients screened, 20 patients were randomized in the study from 4 centers. Of these, 18 patients completed the study and 2 patients (BM-MSC arm) were withdrawn from the study due to fatal SAEs. The SAEs were attributed to the progression of disease.

### Procedural safety

No infection, bleeding, or other complications related to the microbiological condition of the cells were detected in any patient after administration of BM-MSC/placebo which were well tolerated. There were no other procedural related complications like allergic reactions or local swelling because of intramuscular injection of the IMP suggesting that the allogeneic BMMSCs were safe to inject into the CLI patients.

### AEs & SAEs

The total number of adverse events as classified by MedDRA Primary System Organ Class and Preferred Term recorded in the study was 58 (Table 4). There was considerable difference in the overall incidence of AEs between the two arms, with 13 AEs were reported by 6 patients in BM-MSC arm and 45 AEs in 8 patients in the placebo arm (p = 0.6256). None of the AEs in the BM-MSC arm were related to treatment.

**Table 4:** Summary of AEs

	**BM-MSC**	**Placebo**
	**No. of patients**	**No. of patients**
	**(No. of events)**	**(No. of events)**
System organ class		
At least one symptom	6 (13)	7 (45)
Gastrointestinal disorders	1 (1)	2 (2)
General disorders and administration site conditions	3 (3)	0
Hepatobiliary disorders	0	1 (1)
Infections and infestations	3 (3)	3 (3)
Injury, poisoning and procedural complications	1 (1)	1 (2)
Investigations	1 (2)	3 (29)
Metabolism and nutrition disorders	0	1 (1)
Musculoskeletal and connective tissue disorders	0	1 (2)
Nervous system disorders	0	2 (3)
Psychiatric disorders	0	1 (1)
Renal and urinary disorders		1 (1)
Skin and subcutaneous tissue disorders	1 (1)	0
Vascular disorders	1 (2)	0

Most of the AEs observed in the study were due to abnormal clinical laboratory values. One patient in BM-MSC arm and 3 patients in placebo arm developed 2 and 23 laboratory abnormalities respectively. None of the abnormalities were attributed to the IMP administration by the members of the DSMB and the Principal Investigators (PI) of the study where the AEs were observed.

Fourteen SAEs (6 in BM-MSC arm and 8 in placebo arm) were recorded because of hospitalization for disease process related complications. Two patients in BM-MSC arm died during the course of the study: One patient died within seven days of the BM-MSC administration due to cardiac causes as determined by the DSMB and the second patient died after five months of BM-MSC administration due to progression of CLI with ischaemic gangrene leading to amputation and septicaemia. It was concluded by the respective PIs and the DSMB that the cause of the death was not due to IMP administration.

### Laboratory investigations

**Figure 2:**
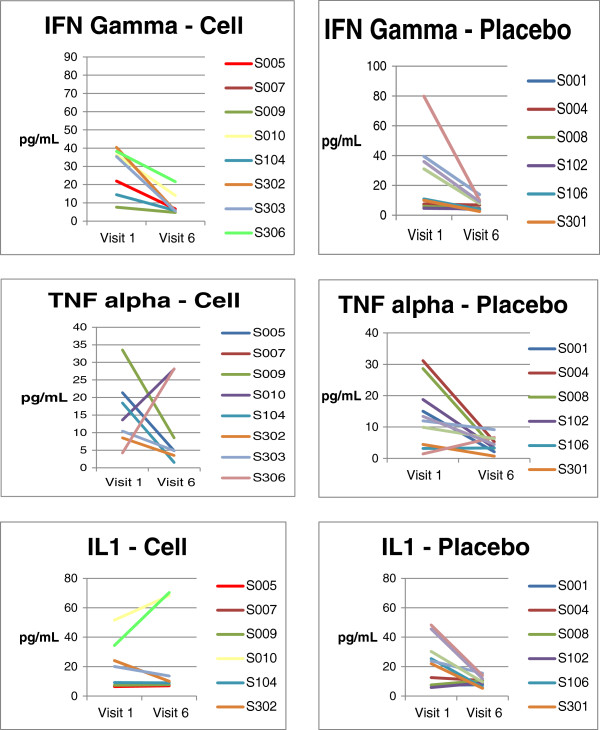
Immunological profile of patients in cell and placebo arm.

Hematological, biochemical and urine analysis results from patients in both treatment arms were comparable at baseline and subsequent visits. Immunological profile (IFN- gamma, IL - 1 & TNF – alpha levels) and lymphocyte profile (CD4, CD8 & CD25) performed at one month, three months and six months after administration of IMP or placebo, revealed that were comparable in both the arms (Figure 2, Tables 5 and 6). No significant difference in the blood lymphocyte profile or in the serum cytokine level was observed between BM-MSC and placebo administered patients suggesting that the administered allogeneic cells did not elicit T-cells proliferative response *in vivo*, as estimated for the values obtained for the various subsets of T lymphocytes (Table 6). With respect to the levels of the pro-inflammatory cytokines we observed a difference between the BM-MSC administered patients and the placebo control patients at baseline, 1 month & 6 months after treatment, but they were either comparable with the baseline values or were within the normal range of these cytokines (Table 5). Collectively, these data indicates that allogeneic BM-MSC administration in CLI patients did not adversely alter the immunological profile.

**Table 5:** Summary of serum Cytokines values at screening, 1 month & 6 month follow-up after BM-MSC implantation

**Test**	**Screening**		**1 month**		**6 months**		**Normal range (pg/ml)**
	**BM-MSC arm**	**Placebo**	**BM-MSC arm**	**Placebo**	**BM-MSC arm**	**Placebo**	
	**N = 10**	**N = 10**	**N = 9**	**N = 10**	**N = 7**	**N = 10**	
	**Mean (SD)**	**Mean (SD)**	**Mean (SD)**	**Mean (SD)**	**Mean (SD)**	**Mean (SD)**	
Gamma - Interferon **(pg/ml)**	24.4(13.35)	23.1(24.07)	37.8 (34.35)	20(24.67)	9.1(6.34)	6.6(3.57)	**0.01 - 168**
Interleukin - 1 **(pg/ml)**	22.6(14.73)	22.9(15.31)	33.1(23.59)	22.9(21.73)	26.6(29.28)	10(3.26)	**0 - 400**
Tumor Necrosis Factor - alpha **(pg/ml)**	14.2(9.16)	13.8(10.11)	16.6(6.16)	15.7(7.98)	11.4(11.65)	4.4(2.47)	**0 – 3.22**

**Table 6:** Summary of lymphocyte profile values at screening, 1 month & 6 month follow-up after BM-MSC implantation

**Test**	**Screening**		**1 month**		**6 months**	
	**BM-MSC arm**	**Placebo**	**BM-MSC arm**	**Placebo**	**BM-MSC arm**	**Placebo**
	**N = 10**	**N = 10**	**N = 9**	**N = 10**	**N = 7**	**N = 10**
	**Mean (SD)**	**Mean (SD)**	**Mean (SD)**	**Mean (SD)**	**Mean (SD)**	**Mean (SD)**
**CD4 (cells/μl)**	**923.1 (217.52)**	**856.3 (237.29)**	**905.6 (134.37)**	**922.4 (451.48)**	**1020.9 (405.67)**	**954.4 (164.49)**
**CD8 (cells/μl)**	**682.5 (253.96)**	**688.3 (420.36)**	**664.2 (183.02)**	**644.9 (396.27)**	**786.3 (329.27)**	**727.9 (312.25)**
**CD25 (cells/μl)**	**21.3 (22.22)**	**16.3 (10.92)**	**21.7 (14.34)**	**22.0 (20.66)**	**30.3 (25.36)**	**35.3 (28.04)**

### Clinical efficacy of allogeneic BM-MSC transplantation

**Figure 3:**
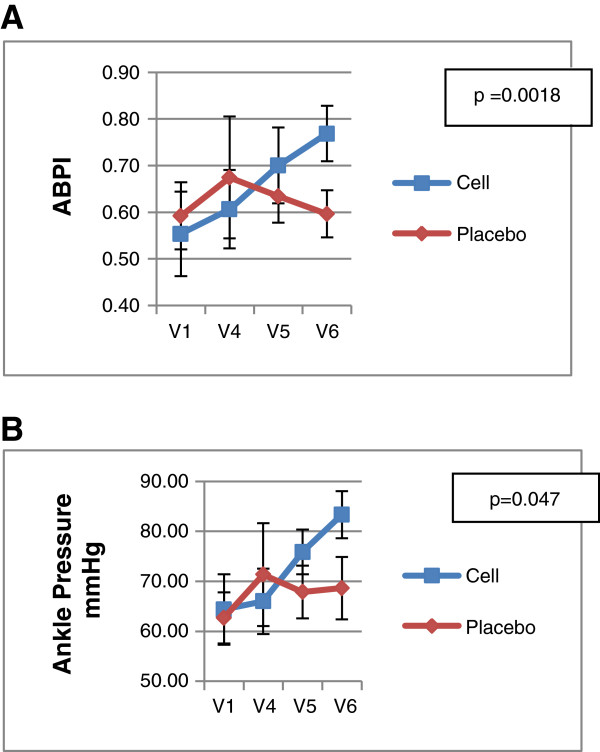
Efficacy parameters: Results of ABPI & Ankle pressure are shown as mean ± SD and corresponding p values. V1: screening; V4: 1 month; V5: 3 month; V6: 6 month follow – up.

1. ABPI & Ankle Pressure: ABPI and ankle pressure were significantly increased at 24 weeks after BM-MSC injection as compared to the values obtained at baseline (Table 7, Figure 3). A mean change of 0.22 was observed in ABPI in BM-MSC arm while there was no change in the placebo arm from baseline to 6 months (p = 0.0018). A mean change of 18.96 mmHg was observed in ankle pressure in the BM-MSC arm compared to 3.92 mmHg change from baseline in the placebo arm (p = 0.047).

**Table 7:** Efficacy parameters

	**BM-MSC arm**		**Placebo**			
	**Baseline**	**6 months**	**Baseline**	**6 months**	**Difference**	**P value**
Rest pain (Median)	3	1	3	0	−1.00	0.1099*
ABPI Mean (SD)	0.554 (0.26)	0.768 (0.15)	0.592 (0.23)	0.596 (0.14)	−0.17	0.0018

*Kruskal-Wallis test.

ANOVA.

2. Rest Pain: Both the groups showed significant improvements in rest pain score from baseline to 6 months (Table 7). The median change from baseline to 6 months in rest pain score of BM-MSC and placebo arm was 2.00 and 3.00 respectively (p = 0.1099).

3. Ulceration: At the time of recruitment 13 patients reported having one or more ulcers, 7 patients in the BM-MSC arm and 6 patients in the placebo arm. At two year follow-up, one patient in the BM-MSC arm continued to have ulcer, however the size of the ulcer decreased, whereas all ulcers healed in placebo arm.

4. Amputation: Four patients (2 in each arm) were identified for toe amputations at screening (pre-planned amputations). During the study, another 5 amputations (three in the BM-MSC arm and two in the placebo arm) were conducted. This included four above knee amputations (2 in each arm), and one toe amputation in the BM-MSC arm. The number and level of amputations were similar in both the treatment arms. The small numbers precluded a reasonable statistical analysis.

## Discussion

To our knowledge, this is the first randomized, double – blinded, placebo controlled multicenter phase I/II trial to assess primarily the safety and also the potential effects of intramuscular allogeneic BM-MSC administration in patients with critical limb ischemia. Our results highlight the safety of using allogeneic bone marrow derived MSCs in patients with CLI and showed positive trends towards improvement as evidenced by the increase in parameters such as ankle pressure and ABPI, consistent with previous reports on cell based therapies in CLI [20-23]. However, patients with impending amputation did not derive any benefit from BM-MSC administration. This group of patients had a poor prognosis, due to the advanced disease process and the clinical outcome was predetermined. For this study, CLI was determined by an ankle pressure of ≤ 70 mmHg along with that of ABPI of ≤ 0.6. Both these parameters showed significant improvement in patients included in the BM-MSC arm while this was not the case in patients treated with placebo (p < 0.05).

Pooled analyses from different studies conducted using stem cells in CLI have shown that ABPI increased between 0.1 and 0.2 points and a TcPO2 increase between 10 and 20 mmHg [24]. The randomized, double – blind, placebo – controlled PROVASA (Intra-arterial progenitor cell transplantation of bone marrow mononuclear cells for induction of neovascularization in patients with peripheral arterial occlusive disease) study showed no significant difference in the primary outcome of improvement in ABPI [25]. However, there were significant improvements in other secondary end points, including ulcer healing and rest pain reduction in the BM-MSC arm group. The authors debated that ABPI was a poor selection as a primary end point. In a recently completed Investigator led trial, we have shown that intra-arterial administration of allogeneic BM-MSCs resulted in significant decrease in pain score (VAS score), ABPI and TcPO2 parameters [26]. It also showed complete ulcer healing to 70% reduction in ulcer area as compared to the baseline values. The TACT trial [23] (therapeutic angiogenesis by cell transplantation) also reported that autologous BM-MNC administration did not alter the ABPI and TcPO2 in patients with atherosclerotic PAD or patients with Buerger’s disease over a period of 3 years, however led to improvement of other efficacy parameters such as extension of amputation free interval and improvement of ischemic rest pain. They concluded that ABPI value is not a useful predictor for evaluating the long – term efficiency of the angiogenic therapy using bone marrow cells. In a previous TACT trial [19] and in our published study [26] ABPI and TcPO2 values were significantly improved in patients with atherosclerotic PAD at 4 and 24 weeks. In another study, Idei et al. [27] reported an increase in ABPI and TcPO2 after BM-MNC implantation in patients with atherosclerotic PAD and Buerger’s disease. In Buerger’s disease, ABPI and TcPO2 were significantly increased after 1 month and remained high during the 3 – year follow – up period. However, in patients with PAD, ABPI and TcPO2 pressure increased significantly after 1 month and gradually decreased during the follow – up, and, returned to the base line values at the end of 3 – year follow – up period. The differences in the severity of PAD may in part explain the observed differences in changes in perfusion between these studies.

We observed decrease in subjective rest pain in all patients irrespective of the arms they belonged to. This may partially be explained by the sufficient concomitant analgesics administered; however, the intake of analgesics was not analyzed objectively. We included patients with diabetes mellitus type 2, which might have caused similar outcomes of rest pain due to the decreased sensitivity of pain with diabetic peripheral neuropathy which was also reported in a study published by Lu et al. [28]. Furthermore, other published studies which showed improvements in rest pain in the BM-MSC arm measured the rest pain score on the Visual Analogue Scale (VAS) of 0 to 10 [20,27] which was not followed in our study.

The adverse events in the BM-MSC arm were less as compared to the placebo. Most of the adverse events were abnormal clinical laboratory values and or symptoms related to the progression of the disease. One patient in the BM-MSC arm died suddenly within one week of IMP administration. DSMB and the Indian FDA did an audit of the patient records, his past history and concluded that the sudden death was not related to IMP and the likely cause of death was a sudden cardiac event, which is not uncommon in patients with CLI [29]. Most studies have shown that cell therapy is promising for angiogenesis and has no severe adverse effects in patients afflicted with Buerger’s disease [19], [30-32]. However, patients with atherosclerotic PAD may have increased mortality due to associated cardiovascular risk factors.

Ulcer healing and amputation rates were similar in both arms in our study. This may be due to the fact that these critically ill patients with impending amputation due to the advanced nature of the disease did not derive much benefit from allogeneic BM-MSC administration. In a study by Walter et al. [25] all 4 patients with extensive gangrene with impending amputation (Rutherford class 6) at inclusion in the study had to undergo amputation above the ankle during the initial 3 month period.

There are few published reports on clinical trial results using autologous or allogeneic BM-MSCs in CLI since majority of the reported trials used autologous BM-MNCs. The mechanism through which MSCs exerts angiogenesis is mainly by secreting angiogenic growth factors or cytokines and also through differentiation into endothelial cells [33,34]. The pro-angiogenic effect of MSC has been demonstrated in several studies both *in vitro* and *in vivo*[33,34]. MSCs have been shown to express and secrete stromal cell-derived factors - 1 (SDF-1), vascular endothelial growth factor (VEGF), basic fibroblast growth factor (bFGF); matrix metalloproteinases (MMPs), all of which are important for triggering and maintaining angiogenesis [35]. However, apart from their angiogenic activity, MSCs obtained from bone marrow and other tissues have also been shown to mediate anti-inflammatory, anti-apoptotic, anti-fibrotic, mitogenic and wound healing properties [36].

Many clinical trials are using autologous BMMNCs for evaluating the efficacy in PAD patients but it has many limitations. Firstly, the active cellular constituent of bone marrow that is the agent of repair is not well characterized. Secondly, it is widely accepted that therapeutically active bone marrow constituents likely represents only 1 in 10,000 bone marrow cells [37]. Thirdly, aspiration of the bone marrow is an invasive process and lastly, concerns exist that patients most likely to be affected by atherosclerosis are also likely to have impaired marrow function [38]. The use of allogeneic BM-MSCs has important advantages. They are likely to represent an enriched population of cells with therapeutic angiogenic capacity. They are readily prepared from healthy donors and may be used as an allogeneic, “off – the - shelf” cryopreserved product [39] without HLA matching because of their hypoimmunogenic, immunosuppressive and immunomodulatory properties.

Although this is a small study performed with a small number of patients (20), it offers some potential clinical insights. As the primary goal of the study, potential safety concerns are alleviated by our findings. Delivery of the BM-MSC via the IM route (in this study) or our earlier published study by IA route [26] did not compromise, rather appeared to have improved the hemodynamic parameters in the lower limbs of the treated patients. This work also forms the basis for future clinical trials aimed at establishing the therapeutic possibility of using allogeneic BM-MSCs in CLI patients to improve angiogenesis and increasing amputation – free survival in these patients.

## Conclusion

This study was conducted to assess the safety of allogeneic BM-MSCs in no – options patients in CLI, and utilized a rigorous double – blind, placebo – controlled, study design. The study met its primary objective of safety with regard to use of allogeneic BM-MSCs for the treatment of CLI. Few of the efficacy parameters showed significant improvements like ABPI and ankle pressure in the BM-MSC arm patients. Our findings support the conduct of more extensive studies with a larger group of patients for assessing the therapeutic efficacy of using allogeneic BM-MSCs for the treatment of vascular disorders.

## Competing interests

Gupta PK, Chullikana A, Das A, Gottipamula S, Krishnamurthy S, Anthony N, Majumdar A S are employees of Stempeutics.

## Authors’ contributions

GPK: Conception and design, administrative support, collection and/or assembly of data, data analysis and interpretation, manuscript writing. CA: Conception and design, collection and/or assembly of data, data analysis and interpretation. PR: Provision of study material or patients. DS: Provision of study material or patients. DA: Conception and design, administrative support, data analysis and interpretation. GS: Manufacturing of Investigational Medicinal product used in this clinical trial. KS: Quality control of the Investigational Medicinal Product used in this trial. AN: Manuscript correction and scientific inputs during finalization of manuscript. PA: Conception and design, data analysis and interpretation, manuscript writing. MAS: Conception and design, data analysis and interpretation, manuscript writing, final approval of manuscript. All authors read and approved the final manuscript.
